# Engineering *Corynebacterium glutamicum* triggers glutamic acid accumulation in biotin-rich corn stover hydrolysate

**DOI:** 10.1186/s13068-019-1428-5

**Published:** 2019-04-15

**Authors:** Jingbai Wen, Jie Bao

**Affiliations:** State Key Laboratory of Bioreactor Engineering, East China University of Science and Technology, 130 Meilong Road, Shanghai, 200237 China

**Keywords:** *Corynebacterium glutamicum*, Lignocellulose hydrolysate, Biotin, Glutamic acid

## Abstract

**Background:**

Lignocellulose biomass contains high amount of biotin and resulted in an excessive biotin condition for cellulosic glutamic acid accumulation by *Corynebacterium glutamicum*. Penicillin or ethambutol triggers cellulosic glutamic acid accumulation, but they are not suitable for practical use due to the fermentation instability and environmental concerns. Efficient glutamic acid production from lignocellulose feedstocks should be achieved without any chemical inductions.

**Results:**

An industrial strain *C. glutamicum* S9114 was metabolically engineered to achieve efficient glutamic acid accumulation in biotin-excessive corn stover hydrolysate. Among the multiple metabolic engineering efforts, two pathway regulations effectively triggered the glutamic acid accumulation in lignocellulose hydrolysate. The C-terminal truncation of glutamate secretion channel MscCG (ΔC110) led to the successful glutamic acid secretion in corn stover hydrolysate without inductions. Then the α-oxoglutarate dehydrogenase complex (ODHC) activity was attenuated by regulating *odhA* RBS sequence, and glutamic acid accumulation was further elevated for more than fivefolds. The obtained *C. glutamicum* XW6 strain reached a record-high titer of 65.2 g/L with the overall yield of 0.63 g/g glucose using corn stover as the starting feedstock without any chemical induction.

**Conclusions:**

Metabolic engineering method was successfully applied to achieve efficient glutamic acid in biotin-rich lignocellulose hydrolysate for the first time. This study demonstrated the high potential of glutamic acid production from lignocellulose feedstock.

**Electronic supplementary material:**

The online version of this article (10.1186/s13068-019-1428-5) contains supplementary material, which is available to authorized users.

## Background

Lignocellulose biomass is the most promising alternative feedstock for the production of glutamic acid that can be used as building block chemical [1]. However, no essential glutamic acid had been successfully produced from lignocellulose biomass until recently the rich biotin content in lignocellulose biomass was identified [2]. The rich biotin in lignocellulose biomass maintains stability during the biorefinery processing and creates an excessive condition for glutamic acid secretion [2]. Excessive biotin resulted in reduced carbon flux for glutamic acid biosynthesis [3] but strengthened cell wall barrier for glutamic acid secretion [2, 4, 5]. Therefore, no glutamic acid can be accumulated by *Corynebacterium glutamicum* cells in biotin-excessive lignocellulose hydrolysate [2]. The general approach to deal with high biotin concentration is the chemical inductions such as adding of β-lactam antibiotics, surfactants or ethambutol [6, 7]. Penicillin and ethambutol addition triggered glutamic acid accumulation in lignocellulose hydrolysate [2, 7]. However, more complicated cell growth and glutamic acid accumulation would be encountered by penicillin or ethambutol induction under the existence of excessive biotin and the typical inhibitors generated from pretreatment step, such as furfural, 5-hydroxymethylfurfural (HMF), and phenolic aldehydes [8]. Thus, only relatively low glutamic acid could be produced by these two triggering methods [2, 7]. Overuse of penicillin and ethambutol also causes environmental and health concerns. Therefore, metabolic engineering of *C. glutamicum* strain to achieve efficient glutamic acid production in lignocellulose hydrolysate is the best way for the production of cellulosic glutamic acid in industrial practice.

Among the genetic modifications to trigger glutamic acid accumulation under excessive biotin condition, knockout of some genes, such as *dtsR* [9], *ltsA* [10], and *odhA* [11], caused glutamic acid accumulation to a certain extent. However, these modifications often resulted in impaired or more susceptible cell growth. Modulating the expression of some lipid synthesis-related genes only led to limited glutamic acid secretion [12]. Therefore, they were not suitable for glutamic acid fermentation in harsh inhibitors containing lignocellulose hydrolysate system. Glutamic acid secretion channel modification is also a potential way to achieve active glutamic acid secretion [13, 14], but its effect on inhibitors containing lignocellulose hydrolysate environment was still unclear. The metabolic engineering of *C. glutamicum* on triggering efficient glutamic acid accumulation from lignocellulose feedstock should balance the glutamic acid secretion from high-biotin-containing hydrolysate and the negative impact of the inhibitors in the hydrolysates on cell growth. In this study, we tried to achieve the glutamic acid accumulation of *C. glutamicum* S9114 in corn stover hydrolysate by the activation of glutamic acid secretion and enhancement of glutamic acid synthesis pathway. Finally, a metabolically engineered *C. glutamicum* XW6 strain suitable for efficient cellulosic glutamic acid accumulation was obtained, and a record-high titer of glutamic acid was achieved using corn stover as feedstock. The engineered strain provides the first practically applicable basis for the production of commodity glutamic acid from lignocellulose biomass for building block chemical use.

## Results

### Triggering glutamic acid secretion by C-terminal truncation of glutamate exporter MscCG

Our previous study showed that the excessive biotin in corn stover completely blocked glutamic acid accumulation in the hydrolysate prepared from pretreated and detoxified corn stover [2]. In this study, we tried two metabolic modifications to facilitate glutamic acid secretion, either by restricting the biotin uptake to reduce the intracellular biotin content to a sub-optimal biotin level suitable for glutamate secretion [15], or by truncating the C-terminal amino acid residue to activate the activity of glutamate exporter MscCG [13, 14].

**Fig. 1:**
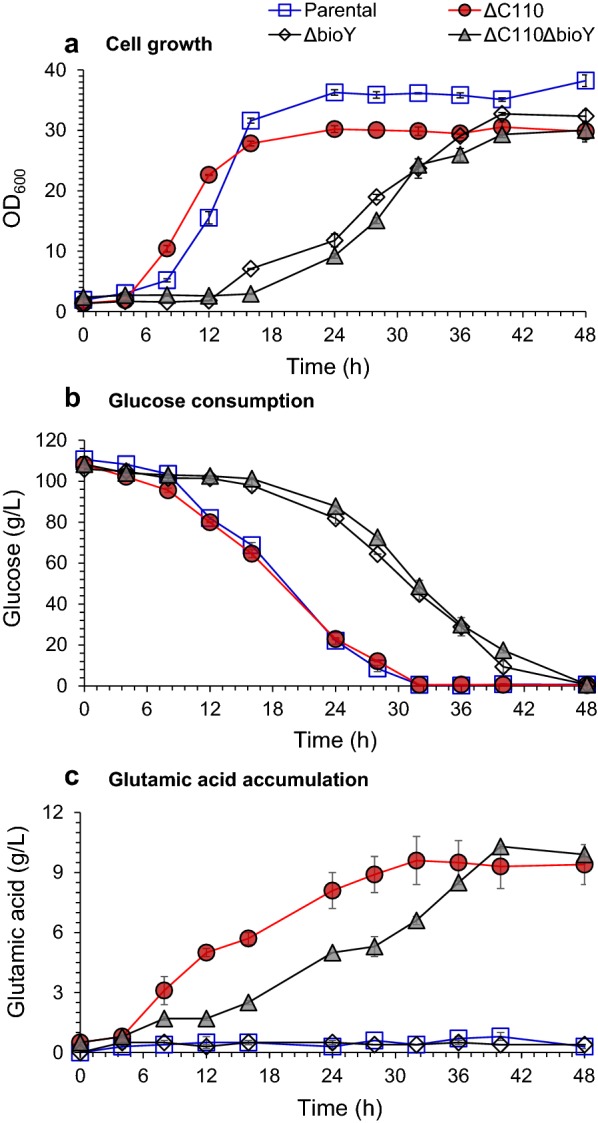
Triggering glutamic acid accumulation by C-terminal truncation of MscCG and *bioY* knockout of *C. glutamicum* S9114. **a** Cell growth; **b** glucose consumption; **c** glutamic acid accumulation. Parental indicates the original *C. glutamicum* S9114 strain; ΔC110 indicates the strain with the MscCG C-terminal truncated; Δ*bioY* indicates the strain with *bioY* gene knocked out; ΔC110Δ*bioY* indicates the strain with the MscCG C-terminal truncated and *bioY* gene knocked out. Glutamic acid fermentations were carried out in 3-L fermentor (3BG-4, Baoxing Biotech Co., Shanghai, China) at 32 °C, 1.4 vvm of aeration and 600 rpm. No penicillin was added for induction. Mean values are presented with error bars representing the minimum and maximum values

To reduce the biotin uptake to reach a sub-optimal level in the cells, we knocked out one of the biotin transporter BioYMN-encoding genes *bioY* by electro-transformation of pK18–Δ*bioY* into *C. glutamicum* S9114 cells following two-round positive selections. Compared to that of the parental *C. glutamicum* S9114, the resulting *C. glutamicum* Δ*bioY* mutant showed longer lag phase time of 12 h (Fig. 1a). The cell growth rate was decreased by about 50%, and the corresponding glucose consumption rate was reduced by about 36% compared to that of *C. glutamicum* S9114 (Fig. 1b). No glutamic acid accumulation was observed in high-sugar corn stover hydrolysate with excessive biotin (approximately 125 µg/L) (Fig. 1c) which was the same as that of *C. glutamicum* S9114. However, glutamic acid was accumulated when culturing the mutant in a less-biotin-containing corn stover hydrolysate (approximately 60 µg/L, but still excessive than the sub-optimal biotin of 2–5 µg/L) (Additional file 1: Figure S1). This result indicates that *bioY* knockout only blocked the biotin uptake partially, and glutamic acid can only be accumulated in low-biotin-containing lignocellulose system. However, high-biotin-containing system still drove sufficient biotin into the cells and failed in glutamic acid accumulation due to the existence of non-specific biotin uptake channel [16]. Therefore, the biotin-dependent glutamic acid secretion cannot be eliminated by *bioY* gene knockout.

For glutamic acid secretion, the *Ncgl1221* gene-encoded glutamate secretion channel protein MscCG was responsible for modulating glutamate export, and its C-terminal region was accountable for the gating process of the channel [13, 14]. The C-terminal 110 amino acid residue truncation achieved glutamate secretion in biotin-excessive conditions without inductions in two different *C. glutamicum* strains [13, 14]. Thus, we tried to truncate its C-terminal residues to activate glutamic acid secretion here. The resulting *C. glutamicum* ΔC110 mutant showed similar cell growth rate with 16% reduced cell mass and similar glucose consumption rate, compared to that of *C. glutamicum* S9114 (Fig. 1a, b). However, glutamic acid accumulation was significantly elevated to 9.6 g/L within 32 h (Fig. 1c). This observed constitutive glutamic acid accumulation indicates the C-terminal truncation of MscCG is an effective method to achieve glutamic acid secretion without inductions in the harsh inhibitors and rich-biotin-containing corn stover hydrolysates. We further integrated the *bioY* knockout with MscCG C-terminal truncation by *bioY* knockout in the MscCG C-terminal truncated ΔC110 strain. The resulting double-knockout-mutant *C. glutamicum* ΔC110Δ*bioY* did not show further significant improvement in glutamic acid accumulation than the single-mutant *C. glutamicum* ΔC110, but the cell growth and glucose consumption rate were reduced to that of the *C. glutamicum* Δ*bioY* mutant (Fig. 1). Since *bioY* knockout was only effective in a relatively low-biotin concentration-containing corn stover hydrolysate, it was reasonable that it did not show any synergistic effect on glutamic acid secretion in the high-sugar corn stover hydrolysate with higher biotin concentration. However, impaired cell growth and glucose consumption would intensely affect glutamic acid productivity. Thus, *bioY* gene knockout in ΔC110 strain was also not suitable for efficient glutamic acid production and *bioY* gene knockout is not an ideal method to trigger glutamic acid accumulation in biotin-excessive corn stover hydrolysate. The effective MscCG C-terminal-truncated ΔC110 strain was used for the subsequent experiments.

### Increasing glutamic acid accumulation by *odhA* attenuation

Decreasing α-oxoglutarate dehydrogenase complex (ODHC) activity is an essential approach to redirect α-oxoglutarate flux to glutamate synthesis instead of succinyl-CoA synthesis [6, 17]. Besides the disruption of *odhA* to completely remove the ODHC activity which blocks TCA cycle and severely inhibits cell growth [11], other methods were also used to decrease ODHC activity, such as changing RBS (ribosome-binding site) [18] or the translational start codon [19], using anti-*odhA* antisense RNA [20] and manipulating *odhA* inhibitory protein OdhI [21–24]. Among these methods, we considered changing RBS sequence as an easier but efficient way, because the enzyme activity can be decreased to certain levels by applying designed RBS sequences with different strength [18, 25]. Thus, the optimal ODHC activity that balances the carbon flux for the TCA cycle and glutamic acid synthesis can be reached. Based on the RBS calculator (https://www.denovodna.com/software/doLogin), the original RBS sequence of *odhA* was evaluated to be 50.38 a.u. (Table 1). Then three RBS sequences with strength of 20 a.u., 10 a.u. and 0.1 a.u. were designed and substituted the original RBS sequence in the genome of the MscCG C-terminal-truncated ΔC110 mutant based on the homologous sacB recombination system [26], and resulted in ΔC110*odhA*RBS20, ΔC110*odhA*RBS10 and ΔC110*odhA*RBS0.1 mutant, respectively.

**Table 1:** RBS sequence and predicted translation initiation rate of *odhA*

Name	Sequence (5′–3′)^a^	Predicted translation initiation rate (a.u.)
Original	CAAGGAAAAGAGGCGAGTACCTGCC	50.38
RBS20	GCTAAATATCATACCGATAAAGTCATA	20.72
RBS10	GGTCCCTCAAGTTAACCACGCGGC	10.33
RBS0.1	CTCACCCACGAGTTCAATAACTAGG	0.11

^a^The strength prediction of original RBS sequence of *odhA* and the design of RBS sequence with different strength are carried out by RBS Calculator (https://www.denovodna.com/software/doLogin)

**Fig. 2:**
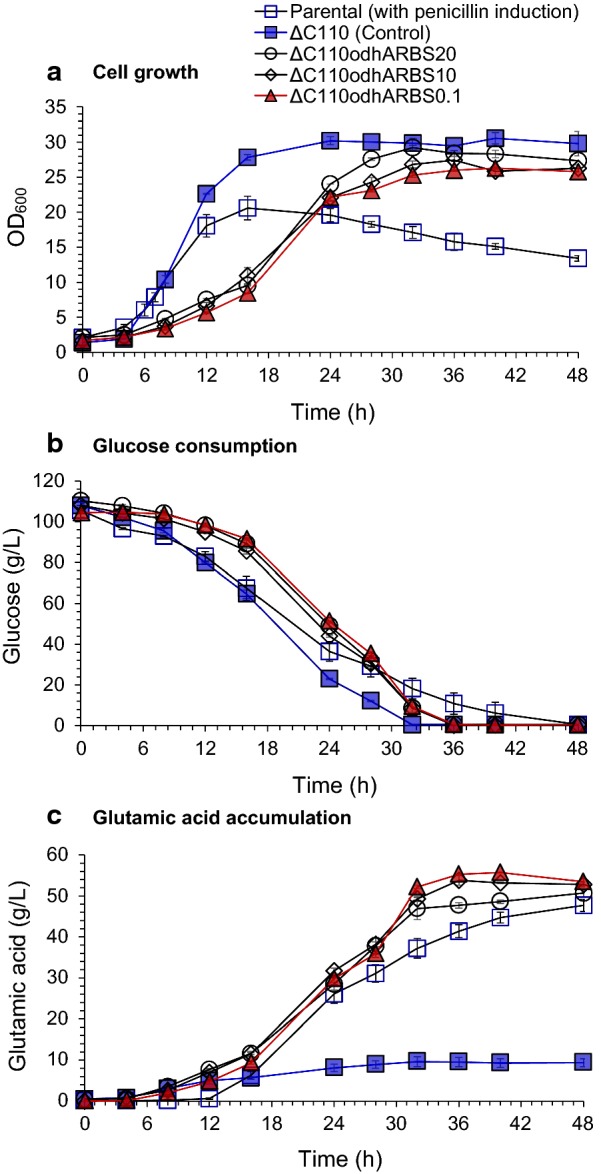
Improving glutamic acid accumulation by adjusting *odhA* RBS strength of ΔC110 strain. **a** Cell growth; **b** glucose consumption; **c** glutamic acid accumulation. Parental (with penicillin induction) indicates the penicillin-triggered glutamic acid fermentation by parental *C. glutamicum* S9114 strain. Penicillin induction method is described in “Methods”. ΔC110 (control) indicates the MscCG C-terminal-truncated strain; ΔC110*odhA*RBS20, ΔC110*odhA*RBS10 and ΔC110*odhA*RBS0.1 indicate the ΔC110 strains with different *odhA* RBS strength of 20 a.u., 10 a.u. and 0.1 a.u. substituted, respectively. Glutamic acid fermentations of engineered recombinants were carried out in 3-L fermentor (3BG-4, Baoxing Biotech Co., Shanghai, China) at 32 °C, 1.4 vvm of aeration and 600 rpm. No penicillin was added for induction. Mean values were presented with error bars representing the minimum and maximum values

The following fermentation assay showed that RBS sequence substitution resulted in normal cell growth during the fermentation with about 30% reduced cell growth rate (Fig. 2a) compared to that of ΔC110 strain, indicating RBS optimization did not deprive the residual ODHC activity for cell growth. Glucose was completely consumed within 36 h compared to 32 h of ΔC110 strain (Fig. 2b). All of the RBS-substituted recombinants exhibited a significant increase in glutamic acid production compared to ΔC110 strain (Fig. 2c). All the three RBS-substituted recombinants produced more than 50 g/L glutamic acid, and the highest glutamic acid titer of 55.7 ± 0.1 g/L and yield of 53.5% within 36 h were achieved by ΔC110*odhA*RBS0.1 strain, which was 5.8-fold higher than that of ΔC110 strain. Thus, decreasing ODHC activity by replacing the original *odhA* RBS sequence with designed RBS with the strength of 0.1 a.u. was suitable for cellulosic glutamic acid production. Higher glutamic acid titer and productivity (16.8% and 55.6% improvement, respectively), as well as more stable cell growth, were achieved compared to that of penicillin-triggered glutamic acid fermentation of *C. glutamicum* S9114. This engineered strain was named as *C. glutamicum* XW6 and used for the following experiments.

### Overexpression of genes on glutamic acid synthesis pathway for accumulation enhancement

To further improve the glutamic acid production, the key genes involved in glutamic acid synthesis from phosphoenolpyruvate to glutamic acid were overexpressed in MscCG C-terminal-truncated and *odhA* gene RBS sequence-substituted *C. glutamicum* XW6 (ΔC110*odhA*RBS0.1) strain. These selected genes include pyruvate carboxylase-encoding gene *pyc* and phosphoenolpyruvate carboxylase-encoding gene *ppc* [27]. Besides, the rate-limiting citrate synthase in the TCA cycle [28]-encoding gene *gltA* and the isocitrate dehydrogenase-encoding gene *icd* [29] were included. We also incorporated two glutamate dehydrogenase-encoding genes *gdh1* and *gdh2*, as well as the modified glutamate exporter-encoding gene. Corresponding overexpression plasmids as well as the empty expression plasmid were electrotransformed into the *C. glutamicum* XW6 cells and then confirmed by colony PCR.

All of the gene overexpression recombinants grew well in the corn stover hydrolysate and produced more than 55 g/L of glutamic acid (Table 2). However, the overexpression of these genes did not result in significant improvement in glutamic acid titer and yield. Compared to that of the control, only 1 g/L higher glutamic acid titer of 60.5 g/L was achieved in the strain with overexpressed *gltA*. This higher glutamic acid titer may be due to the relatively higher initial glucose during the fermentation, and the glutamic acid productivity (1.26 ± 0.02 g L^−1^ h^−1^) and yield (54.0%) were both lower than that of control (1.36 ± 0.11 g L^−1^ h^−1^ and 54.5%, respectively). The modified glutamate exporter overexpression resulted in the same glutamic acid titer, but relatively higher glutamic acid yield (56.1% compared to 54.9%) and 9.6% higher productivity (1.49 ± 0.01 g L^−1^ h^−1^ compared to 1.36 ± 0.11 g L^−1^ h^−1^), compared to that of control. Therefore, overexpression of the modified glutamate exporter would enhance the glutamic acid secretion and benefit the glutamic acid production. We further tried to increase the modified glutamate exporter expression by integrating its encoding genes with H36 promoter to the genome of *C. glutamicum* at CGS9114_RS02700 gene locus which encoded a putative lactate dehydrogenase, and resulted in XW6-ΔRS02700::H36-ΔC110 strain. However, the glutamic acid fermentation of this strain (Additional file 1: Figure S2) did not show any improvement in glutamic acid production performance compared to that of XW6.

**Table 2:** Overexpression of the key genes involving glutamic acid synthesis and secretion on glutamic acid production

Strain^a^	Glutamic acid titer (g/L)	Glutamic acid yield (%)	Glutamic acid productivity (g L^−1^ h^−1^)^b^
XW6-pH36	59.5 ± 0.5	54.9 ± 0.3	1.36 ± 0.11
XW6-pH36–*pyc*	55.3 ± 0.3	50.0 ± 0.8	1.38 ± 0.01
XW6-pH36–*ppc*	55.4 ± 0.4	51.4 ± 1.3	1.39 ± 0.01
XW6-pH36–*gltA*	60.5 ± 0.1	54.0 ± 0.0	1.26 ± 0.02
XW6-pH36–*icd*	58.6 ± 0.0	52.7 ± 1.7	1.22 ± 0.00
XW6-pH36–*gdh1*	56.7 ± 0.7	53.6 ± 0.1	1.18 ± 0.01
XW6-pH36–*gdh2*	55.8 ± 0.4	52.2 ± 1.2	1.40 ± 0.01
XW6-pH36–ΔC110	59.5 ± 0.5	56.1 ± 0.3	1.49 ± 0.01

Glutamic acid fermentations were carried out in 3-L fermentor (3BG-4, Baoxing Biotech Co., Shanghai, China) at 32 °C, 1.4 vvm of aeration and 600 rpm. No penicillin was added for induction

^a^Key genes involving glutamic acid synthesis from phosphoenolpyruvate node and glutamic acid secretion were plasmid-based overexpressed in *C. glutamicum* XW6 (ΔC110*odhA*RBS0.1) strain

^b^Productivities were calculated based on the maximum glutamic acid titer and the time to reach the maximum glutamic acid titer

The other genes’ overexpression even led to lower glutamic acid titer and yield, and no significant improvement in glutamic acid productivity can be reached. Therefore, they had limited effects on improving glutamic acid accumulation here. The important role of pyruvate carboxylase and phosphoenolpyruvate carboxylase in oxaloacetate supply for glutamate production was extensively investigated before [27, 30, 31]. Phosphoenolpyruvate carboxylase often shows severe feedback inhibitions by many metabolic intermediates, while the overexpression of pyruvate carboxylase was more effective in biotin-limited condition because this enzyme requires biotin as co-factor [31]. These may be the reason why no improvement can be achieved by *pyc* and *ppc* overexpression. As for *gltA* and *icd*, their overexpression was supposed to channel more carbon flux to the supply of α-oxoglutarate. However, excessive accumulation of α-oxoglutarate due to the attenuated ODHC activity may offset the effects of *gltA* and *icd* overexpression. The overexpression of the two glutamate dehydrogenases still could not result in improved glutamate accumulation, and this was much the same with the previous report when glutamate dehydrogenase expression level was higher than 13.5-fold in an *odhA*-disrupted mutant [11]. Since H36 was a strong promoter used for high-level gene expression in *Corynebacterium glutamicum* [32], these genes’ overexpression under the control of the strong H36 promoter may not be suitable, and the metabolic burden caused by introducing these expression plasmids should also be taken into consideration. Further investigation should be addressed to show how these genes affect the glutamic acid accumulation in *C. glutamicum* XW6 strain. Just the overexpression of these genes was not effective for improving glutamic acid production in this glutamate-overproducing bacterium. The primary by-product of lactic acid was not formed during all the fermentations of XW6 strain and its derivatives. Therefore, this two-step metabolic-engineered strain (XW6) obtained by activating glutamic acid secretion and attenuating of *odhA* was used for high-titer glutamic acid production in the biotin-excessive corn stover hydrolysate.

### Evaluation of glutamic acid fermentation from corn stover hydrolysate

**Fig. 3:**
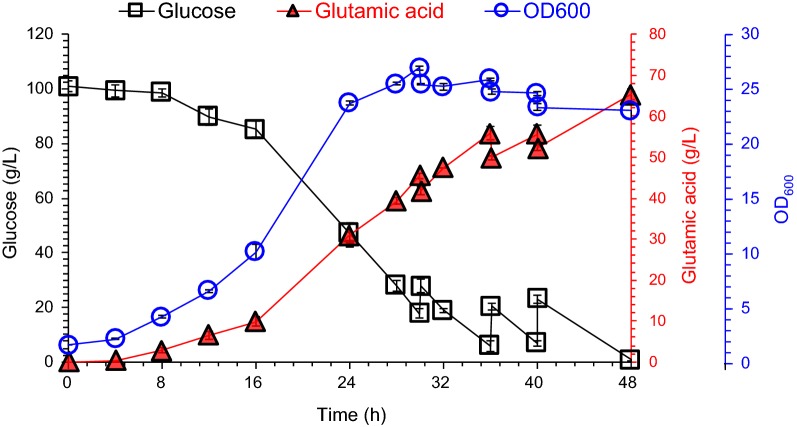
Cellulosic glutamic acid fermentation by the engineered *C. glutamicum* XW6 strain in corn stover hydrolysate. Glutamic acid fermentation was carried out in 3-L fermentor (3BG-4, Baoxing Biotech Co., Shanghai, China) at 32 °C, 1.4 vvm of aeration and 600 rpm. No penicillin was added for induction. The fermentation was initiated by inoculating the seed culture in the 800-mL-corn stover hydrolysate-containing fermentor, and then 125 mL, 150 mL and 175 mL of fresh corn stover hydrolysate was fed into the fermentor at 30 h, 36 h and 40 h, respectively. Mean values are presented with error bars representing the minimum and maximum values

The cellulosic glutamic acid fermentation was conducted for the evaluation of the engineered strain in corn stover hydrolysate. Cells grew well during the fermentation process, cell mass reached a peak at 30 h and maintained at a certain level after the addition of fresh corn stover hydrolysate (Fig. 3). With continuous glucose consumption, glutamic acid continued to accumulate and reached the maximum glutamic acid titer of 65.2 ± 1.4 g/L within 48 h (Fig. 3). Finally, 149.0 ± 1.6 g glucose was consumed and 93.9 ± 2.0 g glutamic acid was produced. The yield based on consumed glucose reached 63.0 ± 0.7%. The glutamic acid titer is record high for glutamic acid production using lignocellulose biomass.

## Discussion

**Fig. 4:**
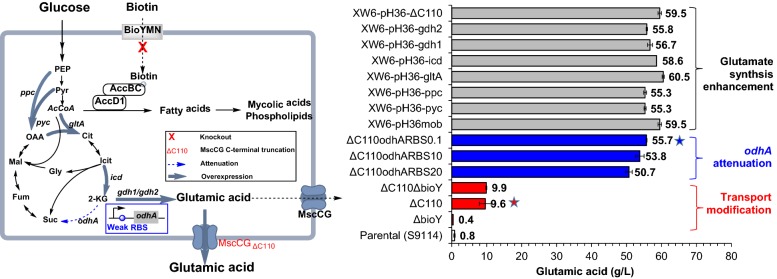
Illustration of *Corynebacterium glutamicum* engineering methods for efficient cellulosic glutamic acid production. The left chart indicates the overall metabolic pathway modifications of *C. glutamicum*; the right chart indicates the elevations of glutamic acid accumulation corresponding to each step of metabolic engineering modification. *PEP* phosphoenolpyruvate, *Pyr* pyruvate, *AcCoA* acetyl-CoA, *Cit* citrate, *Icit* isocitrate, *2-KG* α-oxoglutarate; *Suc* succinate, *Fum* fumarate, *Mal* malate, *OAA* oxaloacetate, *Gly* glyoxylate, *AccBC* acetyl-CoA carboxylase α-subunit, *AccD1* acetyl-CoA carboxylase β-subunit. All the fermentations were carried out in 3-L fermentor (3BG-4, Baoxing Biotech Co., Shanghai, China) at 32 °C, 1.4 vvm of aeration and 600 rpm. No penicillin was added for induction. Mean values are presented with error bars representing the minimum and maximum values

For metabolic engineering of *C. glutamicum* to achieve glutamic acid accumulation under biotin-excessive condition, many reported modifications such as *dtsR* [9], *ltsA* [10], *odhA* [11] knockout often resulted in impaired or more susceptible cell growth. Considering the excessive biotin and harsh inhibitors in lignocellulose hydrolysate, they were not suitable for triggering glutamic acid production in lignocellulose system. Metabolic engineering methods should balance the glutamic acid accumulation and impacts on cell growth. In this study, we tried many methods for metabolic engineering of *C. glutamicum* S9114 for glutamic acid production in biotin-excessive corn stover hydrolysate. Among the methods we carefully screened and tested (Fig. 4), glutamic acid secretion channel modification (C-terminal truncation of MscCG) and *odhA* attenuation by RBS optimization were the most effective ways. These two metabolic modifications successfully triggered glutamic acid accumulation in biotin and inhibitors containing lignocellulose hydrolysate without any penicillin usage. We also tried *bioY* gene knockout and glutamic acid synthesis enhancement, but only slight improvement of glutamic acid accumulation was observed (Fig. 4). Thus, the MscCG C-terminal-truncated and the *odhA* gene RBS sequence-substituted strain XW6 was selected for cellulosic glutamic acid fermentation using corn stover as feedstock after dry acid pretreatment, biodetoxification and enzymatic hydrolysis, and a record-high glutamic acid titer (65.2 g/L) was obtained. Although this glutamic acid titer was still relatively low compared to that of the starch-based glutamic acid fermentation (over 120 g/L) [2], the potential of the engineered strain for cellulosic glutamic acid production using biotin-rich lignocellulose feedstock was fully demonstrated. Future metabolic engineering to co-utilize the lignocellulose-derived pentose sugars and oligomer sugars in lignocellulose hydrolysate such as the xylose, arabinose and cellobiose would further promote the cellulosic glutamic acid production towards industrial application.

## Conclusions

Improved non-chemical-induced glutamic acid production in biotin-excessive lignocellulose hydrolysate was successfully achieved using a metabolic-engineered *C. glutamicum* strain, compared to the penicillin-triggered glutamic acid fermentation by the parental strain. A record high titer of 65.2 ± 1.4 g/L of glutamic acid was produced from corn stover hydrolysate. The biorefinery application of this engineered strain for cellulosic glutamic acid production was fully demonstrated.

## Methods

### Strains, media and culture conditions

Strains and plasmids used in this study are listed in Table 3. *Escherichia coli* DH5α was cultured on Luria–Bertani (LB) medium with 50 μg/mL of kanamycin addition if needed. *C. glutamicum* S9114 was purchased from the Shanghai Industrial Institute of Microorganism (SIIM, http://www.gsy-siim.com/), Shanghai, China, with the storage code of B460. The culture medium was CM2B and contained 10 g/L of peptone, 10 g/L of yeast extract, 10 g/L of NaCl and 10 μg/L of biotin (pH 7.0). The culture condition was 30 °C, 200 rpm, and 50 μg/mL of kanamycin was added if needed. *Amorphotheca resinae* ZN1 was used for biodetoxification, and it was cultured on PDA medium containing 200 g of potato juice, 20 g of glucose and 17 g of agar per liter at 28 °C [8].

**Table 3:** Strains and plasmids

Strains	Characteristics	Sources
*E. coli* DH5α	Host for plasmid construction	Lab stock
*Amorphotheca resinae* ZN1	Biodetoxification fungus isolated by our lab	Lab stock
*C. glutamicum* S9114	Industrial strain for glutamate production	SIIM^a^
Δ*bioY*	In-frame deletion of *bioY* gene	This study
ΔC110	C-terminal truncation of MscCG	This study
ΔC110Δ*bioY*	ΔC110 with in-frame deletion of *bioY* gene	This study
ΔC110*odhA*RBS20	ΔC110 with RBS20 substitution of *odhA* gene	This study
ΔC110*odhA*RBS10	ΔC110 with RBS10 substitution of *odhA* gene	This study
ΔC110*odhA*RBS0.1 (XW6)	ΔC110 with RBS0.1 substitution of *odhA* gene	This study
XW6-pH36	XW6 strain carrying the empty expressing vector	This study
XW6-pH36–*pyc*	XW6 strain carrying the *pyc*-expressing vector	This study
XW6-pH36–*ppc*	XW6 strain carrying the *ppc*-expressing vector	This study
XW6-pH36–*gltA*	XW6 strain carrying the *gltA*-expressing vector	This study
XW6-pH36–*icd*	XW6 strain carrying the *icd*-expressing vector	This study
XW6-pH36–*gdh1*	XW6 strain carrying the *gdh1*-expressing vector	This study
XW6-pH36–*gdh2*	XW6 strain carrying the *gdh2*-expressing vector	This study
XW6-pH36–ΔC110	XW6 strain carrying C-terminal-truncated MscCG expressing vector	This study
XW6-ΔRS02700::H36-ΔC110	XW6 strain with C-terminal-truncated MscCG-encoding gene integrated into the genome at CGS9114_RS02700 locus	This study

Plasmids	Characteristics	Sources
pTRCmob	Overexpression vector, kanamycin resistance Km^R^	[42]
pK18mobsacB	Mobilizable vector, allows for selection of double crossover in *C. glutamicum*, kanamycin resistance, sacB	[42]
pH36mob	Overexpression vector derived from pTRCmob	This study
pK18–ΔC110	Plasmid for truncation of C-terminal of MscCG	This study
pK18–Δ*bioY*	Plasmid for *bioY* gene knockout	This study
pK18–*odhA*RBS0.1	Plasmid for RBS with 0.1 a.u. substitution of *odhA*	This study
pK18–*odhA*RBS10	Plasmid for RBS with 10 a.u. substitution of *odhA*	This study
pK18–*odhA*RBS20	Plasmid for RBS with 20 a.u. substitution of *odhA*	This study
pK18–ΔRS02700::H36ΔC110	Plasmid for integrating C-terminal-truncated MscCG encoding gene into the genome at CGS9114_RS02700 locus	This study
pH36–*pyc*	*pyc* gene-overexpressing vector	This study
pH36–*ppc*	*ppc* gene-overexpressing vector	This study
pH36–*gltA*	*gltA* gene-overexpressing vector	This study
pH36–*icd*	*icd* gene-overexpressing vector	This study
pH36–*gdh1*	*gdh1* gene-overexpressing vector	This study
pH36–*gdh2*	*gdh2* gene-overexpressing vector	This study
pH36–ΔC110	C-terminal-truncated MscCG-encoding gene-overexpressing vector	This study

^a^SIIM indicates the collection center of Shanghai Industrial Institute of Microorganism, Shanghai, China

### Enzyme and reagents

Cellulase Cellic CTec2 was purchased from Novozymes (China), Beijing, China. The filter paper activity was determined to be 203.2 FPU/mL according to NREL protocol LAP-006 [33]; cellobiase activity was determined to be 4900 CBU/mL according to the method reported previously [34]. Total protein concentration was 87.3 mg/mL based on the Bradford method [35]. DNA polymerase and T4 ligase were purchased from Takara, Otsu, Japan. Restriction endonucleases were purchased from Thermo Scientific, Wilmington, DE, USA. Seamless cloning kit HB-infusion^TM^ was purchased from Hanheng Biotech Co., Nanjing, China. Penicillin G with the titer of 1650 U/mg was purchased from New Probe Biochem Co., Beijing, China. Other general chemicals used in this study were of analytical grade and purchased from local suppliers.

### Plasmid and recombinant construction

Plasmids and recombinant strains constructed in this study are listed in Table 3 and the primers used are listed in Additional file 1: Table S1. The upstream and downstream fragments of *bioY* gene were amplified from the genome of *C. glutamicum* S9114 using the primer pairs of bioY-up-F/R and bioY-down-F/R, and subsequently inserted into *Xba*I/*Pst*I and *Pst*I/*Hin*dIII of pK18mobsacB, respectively, resulting in pK18–Δ*bioY*. The upstream and downstream fragments of the corresponding C-terminal 110-amino acid-encoding sequence were amplified from the genome by the primer pairs of ΔC110-up-F/R and ΔC110-down-F/R, and then these fragments were overlapped together by PCR. The fused PCR product was then inserted into *Bam*HI/*Xba*I of pK18mobsacB to generate pK18–ΔC110. For the construction of RBS substitution plasmids of *odhA* gene, three RBS sequences with different strength of 0.1 a.u., 10 a.u. and 20 a.u. were first designed by the RBS calculator (https://www.denovodna.com/software/doLogin). Primer pairs of odhA-up-F/odhA-RBS0.1-up-R and odhA-RBS0.1-down-F/odhA-down-R were used to amplify the upstream and downstream of the original RBS sequence from the genome, and assembled together with *Eco*RI/*Bam*HI-linearized pK18mobsacB fragment by seamless cloning kit, resulting pK18–*odhA*RBS0.1. The same method was applied to construct pK18–*odhA*RBS10 and pK18–*odhA*RBS20 using corresponding primer pairs. For the construction of pK18–ΔRS02700::H36ΔC110, H36 promoter and MscCGΔC110-encoding sequence were amplified from the pH36mob plasmid and *C. glutamicum* S9114 genome, respectively, using primer pairs of H36-F/R and ΔC110-overlap-F/R. Then the up- and downstream of CGS9114_RS02700 were amplified from the genome using primer pairs of RS02700-up-F/R and RS02700-down-F/R. After fused together by overlap PCR using primers of RS02700-up-F/RS02700-down-R, it was assembled together with *Xba*I/*Hin*dIII-linearized pK18mobsacB, resulting pK18–ΔRS02700::H36ΔC110.

The pH36mob plasmid for gene expression used in this study was first constructed by replacing the Trc promoter of pTRCmob with a strong synthetic promoter H36 [32] which was carried out by Shanghai Generay Biotech Co., Ltd, Shanghai, China. The fragment encoding C-terminal-truncated MscCG (MscCGΔC110) was amplified from *C. glutamicum* S9114 genome using primers ΔC110-F/R, and then it was inserted into *Xba*I/*Sal*I restriction site of pH36mob to generate pH36–ΔC110. The fragment of NADPH-dependent glutamate dehydrogenase-encoding gene *gdh1* and another glutamate dehydrogenase-encoding gene *gdh2* was amplified from the genome using primers gah1-F/R and gdh2-F/R and then inserted to *Xba*I/*Sal*I and *Xba*I/*Pst*I of pH36mob, respectively, resulting in pH36-*gdh1* and pH36-*gdh2*. The citrate synthase-encoding gene *gltA* and isocitrate dehydrogenase-encoding gene *icd* were amplified from the genome using primers gltA-F/R and icd-F/R, respectively. Then these two fragments were inserted into EcoRI/XbaI of pH36mob to generate pH36–*gltA* and pH36–*icd*. The pH36-*pyc* plasmid was constructed by ligating the pyruvate carboxylase-encoding gene *pyc* fragment amplified using primers pyc-F/R and the PCR using pH36-vector-F/R-linearized pH36mob together by the seamless cloning kit. Phosphoenolpyruvate carboxylase-encoding gene ppc was amplified from the genome using primers of ppc-F/R, and inserted into *Xba*I/*Sal*I site of pH36mob, resulting in pH36–*ppc*. All the plasmids constructed were sequenced right before use.

Plasmids were transformed into *C. glutamicum* cells by electroporation [36]. The gene disruption, integration and RBS sequence substitution in the genome were based on homologous sacB recombination system [26]. The correct mutants were isolated through two rounds of homologous recombination and confirmed by colony PCR and sequence analysis. Single colonies with kanamycin resistance were picked up and then confirmed by colony PCR using primers pH36-F and corresponding reverse primers of different genes.

### Lignocellulose feedstock and biorefinery processing

Corn stover was harvested in fall 2016, Tongliao, Inner Mongolia, China. The raw biomass was air dried and milled, and then the composition was determined to be 33.0% cellulose, 26.9% hemicellulose, 20.8% lignin and 6.3% ash according to National Renewable Energy Laboratory (NREL) protocols [37, 38]. The dry acid pretreatment was carried out according to our previous procedure [39, 40], and 3.8% (w/w, based on dry material matter) H_2_SO_4_ was used for pretreatment. The pretreated corn stover contained 40.1 mg of glucose, 132.7 mg of xylose, 5.1 mg of furfural, 9.7 mg of 5-hydroxymethylfurfural (HMF), and 19.2 mg of acetic acid per gram of dry corn stover (dry matter, DM). The pretreated corn stover was then biodetoxified according to our previous procedure [8, 41]. Corn stover hydrolysate was prepared by hydrolyzing the pretreated and biodetoxified corn stover material at a solid content of 30% (w/w) dry corn stover solids at a cellulase usage of 10 mg proteins per gram for 48 h at 50 °C. The hydrolysate slurries were centrifuged at 16,125×*g* for 10 min to remove the solid residues and obtain clear supernatant hydrolysate. Then the hydrolysate was autoclaved by a high-pressure steam sterilizer (TOMY XS-700, Tomy Seiko, Co., Tokyo, Japan) at 115 °C for 20 min and filtrated by sterilized filter paper before use. The corn stover hydrolysate was determined to contain 131.6 ± 0.2 g/L glucose, 18.8 ± 0.1 g/L xylose, 2.3 ± 0.0 g/L acetic acid, 0.04 ± 0.01 g/L furfural and 0.02 ± 0.01 g/L HMF.

### Glutamic acid fermentation

*Corynebacterium glutamicum* cells were cultured on CM2B agar at 30 °C for 24–36 h, and then a single colony was picked up for preparing the seed culture as described previously [2]. The batch fermentation was performed in a 3-L fermentor (3BG-4, Baoxing Biotech Co., Shanghai, China) at 32 °C, 1.4 vvm of aeration and 600 rpm. The seed culture was inoculated in 800 mL of corn stover hydrolysate at 10% (v/v) inoculum ratio. The pH was maintained at 7.2 by automatic addition of 25% (w/v) ammonium hydroxide solution. If needed, 50 μg/mL of kanamycin was added to the hydrolysate for maintaining the plasmid. The penicillin-induced glutamic acid fermentation by *C. glutamicum* S9114 followed the same procedure as reported before [2]. All fermentations were carried out in duplicate.

### Analytical methods

Glucose, glutamic acid and lactic acid were analyzed using the SBA-90 biosensor (Biology Institute, Shandong Academy of Sciences, Jinan, Shandong, China). Xylose acetic acid, furfural, and HMF were analyzed on HPLC (LC-20AD, Shimadzu, Kyoto, Japan) equipped with a Bio-rad Aminex HPX-87H column (Bio-rad, USA) and RID-10A detector (Shimadzu, Kyoto, Japan) according to the method reported before [41]. Phenolic compounds were analyzed on HPLC (UV/Vis detector SPD-20A, at 270 nm, Shimadzu, Kyoto, Japan) with a YMC-Pack ODS-A column (YMC Co., Kyoto, Japan) at 35 °C as mentioned before [41]. Biotin concentration in corn stover hydrolysate was determined as reported previously [2]. Cell growth was indicated by optical density at 600 nm (OD_600_) by the UV–visible spectrophotometer BIOMATE 3S (Thermo, Waltham, MA, USA).

### Glutamic acid yield calculation

The glutamic acid yield based on the consumed glucose was calculated by the following equation:

$${\text{Glutamic}} \;{\text{acid}}\;{\text{yield}} = \frac{{\left[ {\text{GMA}} \right] \times V - \left[ {\text{GMA}} \right]_{0} \times V_{0} }}{{\left[ G \right]_{0} \times V_{0} - \left[ G \right] \times V}} \times 100\% ,$$

where [*GMA*] and [*GMA*]_0_ indicate the final and initial concentrations of glutamic acid, respectively, [*G*]_0_ and [*G*] indicate the initial and final concentrations of glucose, respectively, *V*_0_ and *V* indicate the final and initial volumes of the fermentation broth, respectively. The volume change caused by the addition of ammonium hydroxide solution for pH maintenance was ignored in batch fermentation.

## Additional file

**Additional file 1: Table S1.** Primers used in this study. **Figure S1.** Effect of *bioY* gene knockout on glutamic acid accumulation in 15% (w/w) CSH. **Figure S2.** Glutamic acid fermentation by strain XW6-ΔRS02700::H36-ΔC110 in corn stover hydrolysate.

## Abbreviations

CSH: corn stover hydrolysate
HMF: 5-hydroxymethylfurfural
ODHC: α-ketoglutarate dehydrogenase complex
MscCG: mechanosensitive channel
DM: dry matter
NREL: National Renewable Energy Laboratory
SIIM: Shanghai Industrial Institute of Microorganism
CGMCC: China General Microorganism Collection Center
PDA: potato dextrose agar
FPU: filter paper unit
CBU: cellobiase unit
OD_600_: optical density at wavelength (λ) 600 nm
PEP: phosphoenolpyruvate
Pyr: pyruvate
AcCoA: acetyl-CoA
Cit: citrate
Icit: isocitrate
2-KG: α-oxoglutarate
Suc: succinate
Fum: fumarate
Mal: malate
OAA: oxaloacetate
Gly: glyoxylate
AccBC: acetyl-CoA carboxylase α-subunit
AccD1: acetyl-CoA carboxylase β-subunit
