# Supplementary material for: Engineering Corynebacterium glutamicum triggers glutamic acid accumulation in biotin-rich corn stover hydrolysate
Source: Biotechnol Biofuels. 2019 Apr 15;12:86. doi: 10.1186/s13068-019-1428-5 (PMC6463653; doi:10.1186/s13068-019-1428-5)
Supplement: Supplementary file 1 — Additional file 1: Table S1. Primers used in this study. Figure S1. Effect of bioY gene knockout on glutamic acid accumulation in 15% (w/w) CSH. Figure S2. Glutamic acid fermentation by strain XW6-ΔRS02700::H36-ΔC110 in corn stover hydrolysate. [file 13068_2019_1428_MOESM1_ESM.docx]

**Table S1.** Primers used in this study

| **Primers** | **Sequence (5’-3’)** **^*^** | **Restriction enzyme** |
| --- | --- | --- |
| ΔC110-up-F | CGGGATCCCGGGCGCTGCGATTC | BamHI |
| ΔC110-up-R | TCAGCGTCCTATTCCACAGTCATGACCTTAAATAGTGAC |  |
| ΔC110-down-F | TGACTGTGGAATAGGACGCTGATTACAGACGT |  |
| ΔC110-down-R | GCTCTAGATGCATCTGCCACAATATCGC | XbaI |
| bioY-up-F | CTAGTCTAGAAGGATTCTCTCGATTCACCATT | XbaI |
| bioY-up-R | AAAACTGCAGGAAAAAATCATACACCTGAACAGTG | PstI |
| bioY-down-F | AAAACTGCAGATCCAACCCACTTTTCCTCAGA | PstI |
| bioY-down-R | CCCAAGCTTTTTGACCACCGGATAGGGTG | HindIII |
| odhA-up-F | ACAGCTATGACATGATTACGTCAGACGATCCGATCCTAGAAAAC |  |
| odhA-down-R | GCAGGTCGACTCTAGAGCTTGGTCAGACGTGGGACAGAG |  |
| odhA-RBS0.1-up-R | CCTAGTTATTGAACTCGTGGGTGAGCTTCTTGAGGGTTTATTGAGCTTTG |  |
| odhA-RBS0.1-down-F | CTCACCCACGAGTTCAATAACTAGGGTGAGCAGCGCTAGTACTTTCG |  |
| odhA-RBS10-up-R | GCCGCGTGGTTAACTTGAGGGACCCTTCTTGAGGGTTTATTGAGCTTTG |  |
| odhA-RBS10-down-F | CTCAAGTTAACCACGCGGCGTGAGCAGCGCTAGTACTTTCG |  |
| odhA-RBS20-up-R | GCTCACTATGACTTTATCGGTATGATATTTAGCCTTCTTGAGGGTTTATTGAGCTTTG |  |
| odhA-RBS20-down-F | GCTAAATATCATACCGATAAAGTCATAGTGAGCAGCGCTAGTACTTTCG |  |
| RS02700-up-F | GCTCTAGAGACTGCTATCGGTGTGGC | XbaI |
| RS02700-up-R | CCCAGCTTTTGGTAGCCCTTTCAGTTGTTGG |  |
| H36-F | AAAGGGCTACCAAAAGCTGGGTACCTCTAT |  |
| H36-R | GCCTAAAATCATGGATCCCATGCTACTCCT |  |
| ΔC110-overlap-F | AGCATGGGATCCATGATTTTAGGCGTACCCATT |  |
| ΔC110-overlap-R | CAACATTTTTTCTCCCTATTCCACAGTCATGACCTTAAATAG |  |
| RS02700-down-F | TGACTGTGGAATAGGGAGAAAAAATGTTGTCACTCAC |  |
| RS02700-down-R | CCCAAGCTTAGCACTCCTTCAACGCCT | HindIII |
| ΔC110-F | GCTCTAGAATGATTTTAGGCGTACCCAT | XbaI |
| ΔC110-R | ACGCGTCGACCTATTCCACAGTCATGACCTTAA | SalI |
| pH36-Vector-F | GTCCCACCTGACCCCATGCC |  |
| pH36-Vector-R | GCTGAAAATCTTCTCTCATCCGCC |  |
| pyc-F | GAGAAGATTTTCAGCGTGTCGACTCACACATCTTCAAC |  |
| pyc-R | GGGGTCAGGTGGGACTTAGGAAATGACGACGATCAAGTCG |  |
| ppc-F | GGTACCCGGGGATCCTCTAGAATGACTGATTTTCTACGCGATGAC | XbaI |
| ppc-R | CTTGCATGCCTGCAGGTCGACCTAGCCGGAGTTGCGCAGT | SalI |
| gdh1-F | GCTCTAGAATGACAGTTGATGAGCAGGTCTCT | XbaI |
| gdh1-R | ACGCGTCGACTTAGATGACGCCCTGTGCC | SalI |
| gdh2-F | GCTCTAGAATGTTCGAGCTAATCGACGACTG | XbaI |
| gdh2-R | AAAACTGCAGTTATCGGCGAACCATACCTCTG | PstI |
| gltA-F | CCGGAATTCTCACCTATTCCGCTGACAGCTAC | EcoRI |
| gltA-R | GCTCTAGAGGCTGAAATGAGTGGGAGGG | XbaI |
| icd-F | CCGGAATTCACACGTCAGCAATGCGTGG | EcoRI |
| icd-R | GCTCTAGAGCATGTGCCCATTATGGCA | XbaI |
| pH36-F | CAACGCAATTAATGTGAGTTAGCGC |  |
| pH36-R | TCTGTATCAGGCTGAAAATCTTCTC |  |

**^*^** Underlined sequence indicates the corresponding restriction enzyme site.

**Figures**

**Figure S1.** Effect of bioY gene knockout on glutamic acid accumulation in 15% (w/w) CSH. Glutamic acid fermentation was carried out in 3L-fermentor (3BG-4, Baoxing Biotech Co., Shanghai, China) at 32 ^o^C, 1.4 vvm of aeration and 600 rpm. No penicillin was added for induction. Mean values were presented with error bars representing the minimum and maximum values.

**Figure S2.** Glutamic acid fermentation by strain XW6-ΔRS02700:: H36-ΔC110 in corn stover hydrolysate. Glutamic acid fermentation was carried out in 3L-fermentor (3BG-4, Baoxing Biotech Co., Shanghai, China) at 32 ^o^C, 1.4 vvm of aeration and 600 rpm. No penicillin was added for induction. Mean values were presented with error bars representing the minimum and maximum values.
